# Sonographic Measurements of Rectus Femoris Muscle Thickness Strongly Predict Neutropenia in Cancer Patients Receiving Chemotherapy

**DOI:** 10.3390/cancers16051061

**Published:** 2024-03-05

**Authors:** Gürkan Güner, Levent Özçakar, Yusuf Baytar, Mehmet Ruhi Onur, Metin Demir, Burak Yasin Aktaş, Oktay Halit Aktepe, Deniz Can Güven, Hakan Taban, Hasan Çağrı Yıldırım, Serkan Akın, Sercan Aksoy, Murat Kara, Ömer Dizdar

**Affiliations:** 1 Department of Medical Oncology, Hacettepe University Cancer Institute, Ankara 06230, Turkey; dr_metindemir@hotmail.com (M.D.); byaktas@hotmail.com (B.Y.A.); droktayaktepe@hotmail.com (O.H.A.); denizcguven@hotmail.com (D.C.G.); hakantaban@yandex.com (H.T.); hasan-cagri@windowslive.com (H.Ç.Y.); drserkanakin@gmail.com (S.A.); saksoy@hacettepe.edu.tr (S.A.); omer.dizdar@hacettepe.edu.tr (Ö.D.); 2 Department of Medical Oncology, Medical Point Hospital, Izmir Economy University Faculty of Medicine, Izmir 35575, Turkey; 3 Department of Physical and Rehabilitation Medicine, Hacettepe University Faculty of Medicine, Ankara 06230, Turkey; lozcakar@yahoo.com (L.Ö.); mkaraftr@yahoo.com (M.K.); 4 Department of Radiology, Hacettepe University Faculty of Medicine, Ankara 06230, Turkey; bayt93@gmail.com (Y.B.); ruhionur@yahoo.com (M.R.O.)

**Keywords:** malignancy, treatment, toxicity, skeletal muscle mass, ultrasound

## Abstract

**Simple Summary:**

Individuals with cancer often experience a significant incidence of low skeletal muscle mass. This prospective cohort study, conducted between 2018 and 2020 in cancer patients undergoing anthracycline-based chemotherapy, aimed to explore the association between low skeletal muscle mass, measured by computed tomography (CT) and ultrasound (US), and hematologic toxicity. Regional muscle measurements were carried out using US, and hematologic adverse events were graded using the Common Terminology Criteria for Adverse Events (CTCAE) v5.0. Results showed that low rectus femoris (RF) muscle thickness, determined by specific threshold values, significantly increased the incidence of grade ≥ 3 neutropenia. Regression analysis confirmed that low RF muscle thickness independently increased the risk of grade 3–4 neutropenia, irrespective of age, gender, and body mass index. This study suggests that utilizing US for measuring RF muscle thickness can help identify cancer patients at a higher risk of developing neutropenia, enabling more vigilant monitoring and timely implementation of supportive measures in clinical practice.

**Abstract:**

The objective of this study was to explore the possible association between low skeletal muscle mass (SMM)—assessed by computed tomography (CT) and ultrasound (US)—and hematologic toxicity in cancer patients. A prospective cohort study was conducted in cancer patients who received anthracycline-based chemotherapy between 2018 and 2020 and who had baseline abdominal CT including L3 level for measuring SMM. Regional muscle measurements were carried out using US. A total of 65 patients (14 males, 51 females) were included. ROC (receiver operating characteristic) analysis identified threshold values of 18.0 mm [AUC (area under the curve) = 0.765] for females and 20.0 mm (AUC = 0.813) for males, predicting severe neutropenia. Using these cut-offs, females with low rectus femoris (RF) thickness (<18.0 mm) had a significantly higher incidence of grade ≥3 neutropenia (50.0% vs. 10.8%, *p* = 0.005), and males with low RF values (<20.0 mm) had a higher incidence (80.0% vs. 22.2%, *p* = 0.063). A regression analysis, irrespective of age, gender, and body mass index, revealed that only low RF muscle thickness increased the risk of grade 3–4 neutropenia by 9.210 times (95% CI = 2.401–35.326, *p* = 0.001). Utilizing US to measure RF muscle thickness aids in identifying cancer patients at an elevated risk of developing neutropenia. Needless to say, US can serve as a convenient and easily accessible tool for assessing low SMM, providing repeat point-of-care evaluations in clinical practice.

## 1. Introduction

A notable prevalence of low skeletal muscle mass (SMM) exists among individuals with cancer. Furthermore, during the later phases of cancer, most patients demonstrate the presence of low SMM [1,2]. Several investigations have been undertaken to explore the predictive significance of low SMM. Specifically, there has been extensive research into the correlation between low SMM and survival outcomes [2,3,4]. The prognostic significance of low SMM has been established across various cancer types, including lung [3], colorectal [5], breast [6], renal [7], and head and neck cancer [8]. Low SMM has also been explored as a predictive indicator for adverse events, including chemo/radiotherapy toxicity and surgical complications [5,6,7,9,10].

Even though researchers have extensively studied the predictive significance of low SMM, the actual mechanism remains speculative. Various theories propose potential factors contributing to the underlying pathophysiology of low SMM, including age, intracellular oxidative stress, and genetic elements. Additionally, in cancer patients, there is a significant likelihood of experiencing cachexia, which may contribute to the development of low SMM [3,11]. There is a wide literature showing that chemotherapy toxicity increases in patients with low SMM, leading to toxicities (dose-limiting toxicities—DLT) that require dose reduction or delay of treatment [9,12,13,14,15]. Multiple theories exist regarding how low SMM influences toxicity. One hypothesis suggests that the modified ratio of fat-to-lean body mass may impact the pharmacokinetics of anti-cancer drugs [16]. Alternative theories propose that low SMM is autonomously linked to frailty, leading to an increased susceptibility to adverse events [4,17]. The prevailing hypothesis is built on how low SMM affects the distribution of drugs. The human body comprises two primary compartments, fat mass and lean body mass (LBM). Drugs may tend to be distributed preferentially to one of these compartments. Individuals with low SMM typically show a decline in LBM. Since muscle mass constitutes the primary portion of LBM, this can lead to heightened drug concentrations in the plasma, thereby increasing the risk of toxicity [6,8,9,16].

Different methods are utilized to evaluate muscle mass in clinical practice, computed tomography (CT), magnetic resonance imaging (MRI), dual-energy X-ray absorptiometry (DEXA), and bio-impedance analysis (BIA) [18]. DEXA relies on X-rays that decrease in energy according to the composition and thickness of the material they traverse. On the other hand, BIA gauges body composition by utilizing an electrical current, which encounters greater resistance in adipose tissue compared to electrolyte-rich fluids [5,16]. Computed tomography is the technique most commonly employed due to its widespread integration into regular maintenance for the majority of individuals with cancer, coupled with its established high accuracy in measuring SMM. The majority of studies measure SMM by using CT scans at the 3rd lumbar (L3) vertebra level, despite the use of alternative levels [19,20]. In the field of clinical practice, the utilization of these methods is constrained by factors such as their expense, accessibility, and ease of use.

Recently, there has been a growing interest in the use of ultrasound (US) for assessing SMM in clinical settings. It has emerged as a valuable tool for studying low SMM, offering advantages such as affordability, portability, and the absence of ionizing radiation when compared to alternative methods [21], despite the presence of acute and chronic diseases, and disruptions in bodily fluid balance are evident [22], also demonstrating comparability with other methodologies like DEXA, CT, or MRI [23]. A previous investigation established a strong association between quadriceps muscle thickness (MT) measured by US and the isometric maximum voluntary contraction force. This finding suggests that US can serve as a viable technique for assessing low SMM [24]. Further, the measurement of MT using US, whether at a single or multiple body sites, is frequently linked to SMM [25]. Low SMM does not manifest uniformly; it disproportionately impacts postural muscles compared to non-postural ones [26]. Hence, it may be necessary to conduct site-specific evaluations to detect the early signs of muscle mass loss accurately. In line with this, recent research indicates that US-based assessment of muscle thickness can identify thigh low SMM before it becomes apparent at the whole-body level [27,28]. Minetto et al. [29] reported site-specific cut-points for US-based detection of low SMM, which are 20 mm in men and 16 mm in women for rectus femoris (RF) muscle thickness.

This study aimed to investigate the potential relationship between low SMM and hematologic toxicity in cancer patients by evaluating skeletal muscles with CT and US before the onset of chemotherapy.

## 2. Materials and Methods

### 2.1. Patients

A prospective cohort study was conducted in cancer patients who received anthracycline-based chemotherapy at Hacettepe University Hospital between 2018 and 2020. The participants were individuals aged 18 years and above, newly diagnosed with cancer, and scheduled to undergo chemotherapy. Patients who had a baseline abdominal CT containing L3 level for measuring SMM were included. Regional muscle thicknesses were measured using US. The study was conducted in accordance with the Declaration of Helsinki and approved by the Institutional Review Board of Hacettepe University (GO 18/536-38, 9 October 2018). Informed consent was obtained from all participants.

### 2.2. Data Collection

Patient demographics, comorbidities, underlying cancer diagnosis, chemotherapy regimen, anthracycline type and doses, and chemotherapy toxicities were collected for each patient. Records of baseline (before chemotherapy) and follow-up laboratory values were also documented.

### 2.3. Measurement of Skeletal Muscle Area and Definition of Low Skeletal Muscle Mass

#### 2.3.1. CT Measurements

To evaluate low SMM, we used the SMM at the L3 vertebra. CT examinations were acquired with 0.6 mm or 2 mm slice collimation thickness at multidetector CT equipment (Siemens SOMATOM Sensation, Siemens SOMATOM Perspective, Siemens Healthineers, Erlangen, Germany, GE Optima 540, General Electric Company, Boston, MA, USA). The reconstructed CT slices with 5 mm thickness, aligned parallel to the end plates of the L3 vertebra and crossing the midline of the L3 vertebral body, were used to calculate the total volume of skeletal muscle for a 1 cm thickness. Acquired CT images were transferred to a workstation (Syngo.via VB60A_HF07 Software, Siemens Healthcare^®^, Erlangen, Germany). The ellipsoid contours of the abdomen were selected manually and muscle density threshold values (−30–150 HU) were defined on the software to confine muscle areas. After selecting threshold HU values, a dedicated software in the workstation revealed the area values of skeletal muscle tissues on the selected image section automatically. Area values of skeletal muscles were multiplied with section thickness ratio to obtain volume value at 1 cm^3^ section thickness.

#### 2.3.2. US Measurements

Regional muscle thicknesses (anterior/posterior upper arm, abdomen, subscapular region, anterior/posterior thigh, anterior/posterior lower leg) were measured using a 5–12 MHz linear probe (Logiq P5, General Electrics, Kaukauna, WI, USA) [30,31,32,33]. US images were acquired without compression, using an ample amount of gel, while subjects were positioned both in supine and prone postures. Muscle measurements were taken at the same axial point. A single physiatrist, with 25 years of experience in musculoskeletal US, conducted all measurements on the dominant side of the body.

### 2.4. Definition of Myelotoxicity

To evaluate myelotoxicity, we conducted repeat blood tests of hematological parameters (complete blood count and biochemistry) at baseline, before each therapy cycle, and 1–3 weeks after each cycle. The frequency of anemia, neutropenia, and thrombocytopenia was calculated as the percentage of patients who developed each toxicity during chemotherapy. The severity of hematologic adverse events was graded using the Common Terminology Criteria for Adverse Events (CTCAE) v5.0 [34], and toxicities graded as ≥3 were considered significant.

### 2.5. Statistical Analysis

Statistical analyses were performed using SPSS software version 22 (SPSS Inc., Chicago, IL, USA). First, the variables were assessed for normality using visual (histogram, probability plots) and analytical methods (Shapiro–Wilks test). Descriptive statistics are presented as mean ± standard deviation for normally distributed variables and as median (IQR) for skewed distributed variables. Categorical variables are reported as frequencies and percentages. Comparisons between groups were performed using one-way ANOVA or Kruskal–Wallis tests (depending on the normality for numerical variables) and using Chi-square and Fisher’s exact tests for categorical variables.

In the univariate analysis, age, body mass index (BMI), and rectus femoris (RF), vastus intermedius (VI), vastus lateralis (VL), and gastrocnemius muscles’ thicknesses (measured by US) were found to be significant among the groups. Since there were high correlations among muscle thickness variables, they were analyzed one by one to avoid collinearity, and it was observed that the most significant result was found with RF thickness. It was observed that there was no independent relationship when using CT-derived measurements in the regression analyses. Additionally, as there was a high correlation between BMI and BSA, the more meaningful variable (i.e., BMI) was included in the analysis. In addition, although gender was not significant, it was included in the analysis since it was a clinically important variable. Thus, the most statistically significant models were presented as the final models.

**Figure 1:**
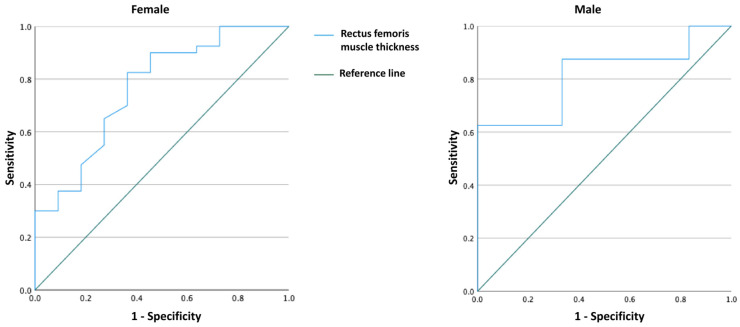
Receiver operating curve of rectus femoris muscle thickness for predicting CTCAE grade 3–4 neutropenia. CTCAE: Common Terminology Criteria for Adverse Events.

The best cut-off values of the US-derived RF measurements for the prediction of CTCAE grade 3–4 neutropenia were found through maximum Youden’s index, using the receiver operating characteristic (ROC) curve for each gender (Figure 1). The sensitivity and specificity measured by the area under the curve (AUC) were used to compare the overall diagnostic accuracy among the predicted models. For the multivariate analysis, the possible factors (i.e., age, gender, BMI, and having low (RF) thickness) identified with univariate analyses were further entered into the binary logistic regression analysis (with enter method) to determine independent predictors of patient outcome (i.e., CTCAE grade 3–4 neutropenia). The Hosmer–Lemeshow test and adjusted R^2^ measures were used for goodness-of-fit of logistic regression models. A *p* value < 0.05 was considered statistically significant.

## 3. Results

A total of 65 patients (14 male, 51 female) were included; 40 (61.5%) with breast cancer, 17 (26.2%) with lymphoma, and 8 (12.3%) with sarcoma. The mean age of the patients was 46.3 ± 14 years, the mean BMI was 27.4 ± 4.9 kg/m^2^, and the mean BSA was 1.82 ± 0.17 m^2^. Their demographic and clinical characteristics are presented in Table 1. A comparison of the clinical variables according to neutropenia is presented in Table 2.

According to the ROC analysis, 18.0 mm (AUC = 0.765 [95% CI = 0.602–0.927]) in females and 20.0 mm (AUC = 0.813 [95% CI = 0.578–1.000]) in males were determined as threshold values for RF thickness to predict CTCAE grade 3–4 neutropenia. Considering these cut-off values, females with low RF values (<18.0 mm) had a higher incidence of grade ≥ 3 neutropenia (50.0% vs. 10.8%, *p* = 0.005), and males with low RF values (<20.0 mm) had a significantly higher incidence of grade ≥3 neutropenia (80.0% vs. 22.2%, *p* = 0.063) (Table 3). When regression analysis was performed according to the low RF muscle thickness; only low RF muscle thickness increased the development of grade 3–4 neutropenia by 9.210 times (95% CI = 2.401–35.326)—regardless of age, gender, and BMI (*p* = 0.001) (Table 4). When the analysis was repeated only in patients with breast cancer (N = 40), only low RF thickness increased the development of grade 3–4 neutropenia by 6.629 times (95% CI = 1.283–34.238)—regardless of age, and BMI (*p* = 0.024) (Table 5).

## 4. Discussion

In this study, our objective was to examine the potential correlations between low SMM (measured by CT and US) and hematologic toxicity in cancer patients before the onset of chemotherapy. We found that US measurement of the RF muscle might be the most precise/convenient approach for predicting hematologic toxicity in these patients. Prior research has demonstrated a correlation between low SMM and chemotherapy-induced toxicity, spanning various chemotherapy protocols and cancer types [5,6,8,9,35]. However, similar analyses using US criteria has not been undertaken in cancer patients. Therefore, to our best notice, this prospective clinical study represents the first data in the hitherto literature.

Based on our results, low SMM could serve as an indicator for predicting hematologic toxicity in individuals undergoing chemotherapy. Hematologic toxicity appears to be intimately linked with skeletal muscle atrophy in cancer patients. Excessive toxicity in individuals with diminished SMM is a result of the usual practice of chemotherapy dosing based on a patient’s height and weight, and consequently body surface area, without taking into account that a substantial and unpredictable portion of body weight comprises fat mass [36]. It is essential to remember that the body surface area (BSA) is a conventional method, initially calculated by Du Bois and Du Bois [37] over a century ago, for calculating doses during the administration of cytotoxic chemotherapy. Body composition, which includes the amount and distribution of fat and lean soft tissue, is a factor that affects the pharmacokinetics and metabolism of many chemotherapeutic agents. Previous studies have shown significant differences in body composition among patients with the same BSA. Reduced muscle mass may lead to a decreased tissue volume for the dispersion of specific cancer treatments, potentially causing diminished metabolism and drug clearance capabilities and, eventually, an elevated risk of toxicity [38,39]. In addition, Prado et al. [40] have shown that fat-free mass, indicative of the distribution volume for numerous cytotoxic chemotherapy drugs, exhibits a weak correlation with body surface area in obese individuals with cancer. The diversity in fat-free mass among individuals can explain the variability in the effective distribution volume for chemotherapy given per unit of body surface area, which may be up to three times. All of these observations underscore the connection between low SMM and hematologic toxicity. As a result, we administer chemotherapy medication doses based on BMI, but alternative dosing may be necessary for improved efficacy and side-effect control.

In our study, we assessed 65 cancer patients using both US and CT scans to establish the relationship between low SMM and hematologic toxicity. In binary logistic regression analyses, only low RF muscle thickness increased the development of grade 3–4 neutropenia by 9.210 times (95% CI = 2.401–35.326), regardless of age, gender, and BMI. In the relevant literature, the thickness of RF or quadriceps muscle has been suggested as a valuable indicator for the early and precise identification of muscle mass loss [27,29,41]. This study has also demonstrated that utilizing US-derived parameters of the RF muscle is a more precise way of predicting low SMM and myelotoxicity, compared to the CT measurement of L3 SMM.

The loss of skeletal muscle is not a uniform condition, impacting postural muscles more significantly than non-postural ones [42,43,44,45]. Consequently, a site-specific assessment for the early/accurate detection of muscle mass loss may be necessary. Indeed, sonographic identification of thigh muscle mass loss is possible before the detection of overall skeletal muscle loss [27,45]. This would also imply that the sonographic measurement of muscle mass loss can be comparable to other methods, e.g., CT or MRI [46]. Consistently, recent studies have shown that US-based assessment of muscle thickness can detect the loss of skeletal muscle in the thigh before it becomes apparent at the whole-body level [27,28]. Likewise, our results indicate that site-specific thigh muscle loss may manifest before it becomes discernible at the whole-body level.

Thigh muscle loss in specific areas challenges the idea of uniform age-related muscle loss. Lifelong physical activity intensity and duration, along with age-related neuromuscular changes, may reduce activation of anterior muscles. Research supports this, showing declines in motor units at specific sites with increasing age [47]. Furthermore, reduced androgen concentrations associated with aging may also contribute to site-specific skeletal muscle mass loss. Hormone receptors exhibit increased activity in actively exercising muscles but not in muscles with lower levels of physical activity. As a result of potential localized decreases in muscle activation, there might also be a decline in hormonal binding within the anterior region of the thigh [48]. Insulin resistance is another factor in site-specific muscle loss. Men with insulin resistance experience more significant muscle mass loss with age compared to insulin-sensitive individuals [49], and different muscle groups, like type IIb fibers, may show varied responses [50]. Ultimately, muscle microstructure changes with age, similar to disuse-induced atrophy. For instance, disuse atrophy affects antigravity muscles more than their counterparts (e.g., quadriceps more than hamstrings). Moreover, aging-related structural changes may maintain anterior thigh muscles at a shortened length, linked to a decrease in sarcomeres [51]. In the future, research may elucidate the molecular mechanisms linked to the decline in muscle mass at specific sites.

The limitation of our study was Its relatively small sample size. The patient cohort was diverse, encompassing individuals with different types of cancers at both early and advanced stages.

## 5. Conclusions

Sonographic measurement of RF muscle thickness provides an easy/objective tool to assess SMM and can assist in early identification of cancer patients who are at high risk for developing neutropenia. For sure, repeat measurements during daily clinical point-of-care can enable more vigilant monitorization of side effects as well as timely initiation of supportive measures.

## Figures and Tables

**Table 1:** Demographic and clinical characteristics of the patients (N = 65).

Characteristic	
Age, years	46.3 ± 14
Gender, female	51 (78.5)
Diagnosis	
Breast	40 (61.5)
Lymphoma	17 (26.2)
Sarcoma	8 (12.3)
Comorbidities	
Hypertension	7 (10.8)
Diabetes mellitus	3 (4.6)
CAD	2 (3.1)
COPD	1 (1.5)
Asthma	3 (4.6)
Hypothyroidism	2 (3.1)
BMI, kg/m^2^	27.4 ± 4.9
BSA, m^2^	1.82 ± 0.17
Laboratory test	
Hemoglobin, g/dL	12.6 ± 1.7
MCV, fL	83.4 ± 6.3
RDW, %	14.1 (13.5–15.5)
Albumin, g/dL	4.1 (3.8–4.4)

Data are given as n (%), mean ± SD, or median (IQR); CAD: coronary artery disease; COPD: chronic obstructive pulmonary disease; BMI: body mass index; BSA: body surface area; MCV: mean corpuscular volume; RDW: red cell distribution width.

**Table 2:** Comparison of the clinical variables according to neutropenia (N = 65).

	CTCAE Grade 3–4 Neutropenia	CTCAE Grade 1–2 Neutropenia	Normal	*p*
N	17	21	27	
Age	41.9 ± 15.1	43.1 ± 12.4	51.5 ± 13.4	0.037
Gender, female	11 (64.7)	17 (81.0)	23 (85.2)	0.259
BMI, kg/m^2^	26.3 ± 4.9	25.8 ± 4.3	29.5 ± 4.7	0.014
BSA, m^2^	1.81 ± 0.20	1.78 ± 0.18	1.87 ± 0.13	0.191
Adriamycin dosage	420 (388–490)	400 (370–495)	440 (400–480)	0.319
Diagnosis				0.370
Breast	9 (52.9)	11 (52.4)	20 (74.1)	
Lymphoma	5 (29.4)	8 (38.1)	4 (14.8)	
Sarcoma	3 (17.6)	2 (9.5)	3 (11.1)	
US-MT				
Anterior arm	25.4 ± 5.7	24.3 ± 4.1	27.3 ± 4.8	0.104
RA	9.6 ± 2.0	9.5 ± 1.9	9.9 ± 1.4	0.613
RF	17.6 ± 3.4	20.0 ± 3.7	21.8 ± 3.6	0.002
VI	15.7 ± 4.1	16.6 ± 2.9	19.5 ± 3.4	0.001
VL	21.4 ± 2.9	22.7 ± 4.0	24.7 ± 3.1	0.017
Gastrocnemius	17.8 ± 1.9	18.6 ± 1.8	19.5 ± 1.6	0.020
FL	33.7 ± 6.1	34.2 ± 3.6	33.9 ± 3.5	0.975
PA (°)	29.3 ± 4.9	33.3 ± 4.9	31.9 ± 3.8	0.088
CT-L3 (CSA)				
Paravertebral	44.9 ± 10.1	43.3 ± 8.0	47.9 ± 7.9	0.165
Psoas major	18.6 ± 8.3	17.7 ± 5.6	18.9 ± 7.7	0.902
Total	126.9 ± 29.0	124.6 ± 27.5	134.7 ± 28.5	0.278

Data are given as *n* (%), mean ± SD, or median (IQR); CTCAE: Common Terminology Criteria for Adverse Events; N: number; BMI: body mass index; BSA: body surface area; US: ultrasound; RA: rectus abdominis; RF: rectus femoris; VI: vastus intermedius; VL: vastus lateralis; FL: fascicle length; PA: pennation angle; CT: computed tomography; L3: 3rd lumbar vertebra; CSA: cross-sectional area; MT: muscle thickness.

**Table 3:** Fisher’s exact test results.

	CTCAE Grade 3–4 Neutropenia	*p*
Females		0.005
Low RF (*n* = 14)	7 (50.0)	
Normal RF (*n* = 37)	4 (10.8)	
Males		0.063
Low RF MT (*n* = 5)	4 (80.0)	
Normal RF (*n* = 9)	2 (22.2)	

Data are given as *n* (%); CTCAE: Common Terminology Criteria for Adverse Events; RF: rectus femoris, MT: muscle thickness.

**Table 4:** Binary logistic regression analyses for predicting CTCAE grade 3–4 neutropenia (N = 65).

	OR	CI	*p*
Age	0.977	0.926–1.031	0.395
Gender, male *	2.830	0.641–12.501	0.170
BMI	1.029	0.885–1.197	0.711
Low RF MT	9.210	2.401–35.326	0.001

CTCAE: Common Terminology Criteria for Adverse Events; OR: odds ratio; CI: 95% confidence interval; BMI: body mass index; RF: rectus femoris; MT: muscle thickness; * male vs. female; Hosmer–Lemeshow test *p*-value = 0.963. R^2^ value of the regression model = 0.320.

**Table 5:** Binary logistic regression analyses for predicting CTCAE grade 3–4 neutropenia in breast cancer patients (N = 40).

	OR	CI	*p*
Age	0.978	0.897–1.065	0.606
BMI	1.023	0.851–1.230	0.810
Low RF MT	6.629	1.283–34.238	0.024

CTCAE: Common Terminology Criteria for Adverse Events; OR: odds ratio; CI: 95% confidence interval; BMI: body mass index; RF: rectus femoris; MT: muscle thickness; Hosmer–Lemeshow test *p*-value = 0.410; R^2^ value of the regression model = 0.196.

## Data Availability

Data are available upon request.
